# Mitochondrial avatars for quantitative aging research

**DOI:** 10.18632/aging.101409

**Published:** 2018-03-30

**Authors:** Timothy E. Hoffman, Lyle E. Wallis, William H. Hanneman

**Affiliations:** 1 Center for Environmental Medicine, Colorado State University, Fort Collins, CO 80523, USA

**Keywords:** aging, mitochondria, stress responses, computational, simulation

As the search for determinants of degenerative aging continues, research efforts are becoming more focused on molecular targets and pathways that converge on the mitochondrion. The genesis of mitochondrial aging research stems from the original free radical theory of aging, where reactive oxygen species produced spontaneously within each cell’s mitochondria go on to damage the cell in a deleterious feedback loop [1]. This classical theory, albeit rooted in fine physicochemical logic, neglects to include the profoundly robust interconnectivity of living systems, and in doing so, assumes that cells are not built with some level of resilience against this spontaneous oxidative damage.

It is now well understood that cells have many defense mechanisms in place to respond to oxidative damage, especially that of the age-dependent mitochondrial variety [2], but the slew of pathways involved in producing and mitigating damage continues to become more abundant and more highly connected as we reveal more experimental information. One such connection at the forefront of aging research is the relationship between broad spectrum mitochondrial stress and the activation of the mitochondrial unfolded protein response, a story about the complex maintenance of mitochondrial integrity and cellular proteostasis [2]. Intertwined further into these cellular networks are the activities of sirtuins, a type of deacetylases with a multitude of beneficial functions that preserve mitochondrial efficiency and curb age-dependent oxidative signaling [3].

**Figure 1:**
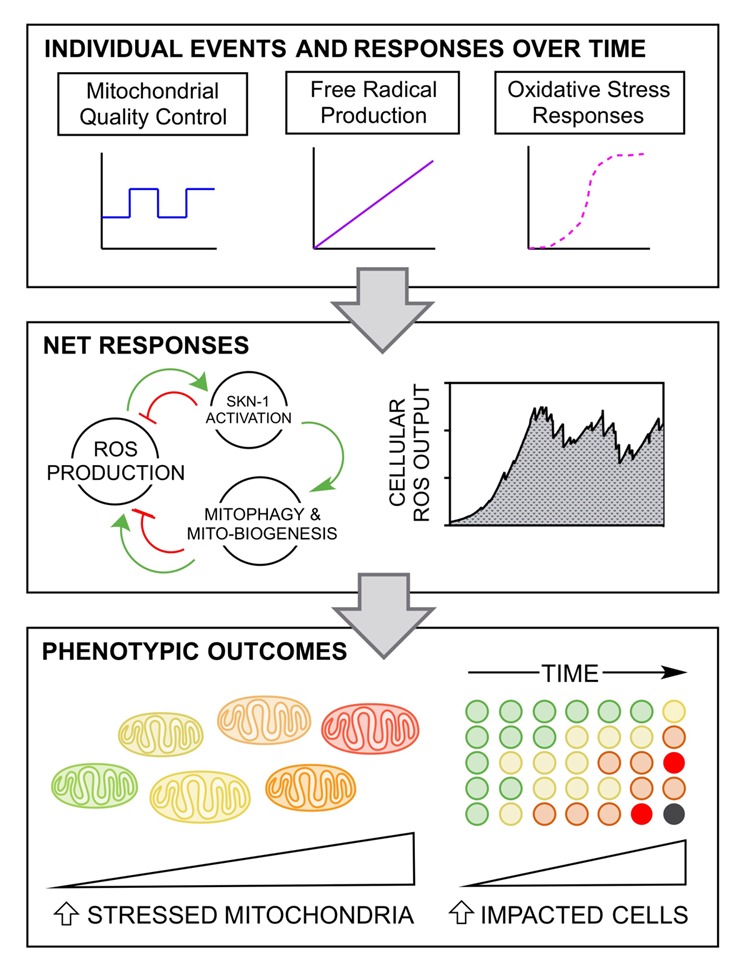
**Quantitative responses compounded to produce the net effects of aging.** The experimental simulation paradigm defined in this perspective describes a computational landscape that quantifies individual cellular responses (top panel), makes use of them in large multifaceted circuits to calculate net responses over time (middle panel), and ultimately determines the phenotypic viability outcomes for both mitochondrial and cell populations.

While it is difficult to identify all the relevant mechanistic details that account for mitochondrial aging, investigators are beginning to approach a realistic comprehensive understanding of the fundamental circuitry at play. The difficulty in this work then rises exponentially as we attempt to quantify the individual cellular circuit components under a variety of environmental conditions and over relevant lengths of time. The purpose in doing so is to quantitatively evaluate these connections and to see their net response when compounded (Figure 1). In this process, a legitimate set of numbers with useful units will surface, and if correct computational methods are employed, you will reveal a focal point that the system depends upon: a node of interest or even a particular set of connections.

In this perspective, we emphasize that the degenerative aging response is not a consequence of a single concrete cellular pathway, but is a phenomenon driven by multifarious events in concert. This is interpreted at the computational simulation interface as the specific magnitude of selected mitochondrion-oriented interactions, often represented by sets of differential equations or discrete numerical events. While simulation techniques robustly complement experimental data and offer us the most hope for interpreting this biogerontological system, scientists continue to search for (i) the most appropriate and insightful computational formats, and (ii) the key cellular network connections and molecular convergences that truly matter [4]. A prime example of this ambitious undertaking is a recent study carried out by Tam et al. (2015) that presents a unique computational approach to understanding a relevant link explaining age-associated damage and dysfunction: the accumulation of faulty mitochondrial contents as a result of organelle fusion-fission dynamics [5]. These computational biochemists successfully constructed a chemical master equation framework to quantitatively demonstrate how mitochondrial dynamics may influence the clonal expansion of aberrant mitochondrial DNA, while implementing expected cellular stochasticity. This study highlights not only how age-dependent damage can occur by simulating a substantial set of mitochondrial processes; it also draws attention to the most significant processes that dictate a cell’s age-dependent outcome.

There is no doubt that it is immensely valuable to quantitate the net effects of molecular pathways to determine age-dependent behavior of a cell; however, the usefulness of this approach is amplified further when it is applied to populations of cells in order to determine the response of whole tissues. Our recent article in Aging Cell [6] attempts to create a similar expansive hierarchical computational landscape, (i) by drawing upon many previous computational models of mitochondrial aging [4,5] and (ii) by building new integrated simulation constructs for significant pathway-driven concepts as they have been identified in the model organism *Caenorhabditis elegans* [2,3]. What our group illuminated was a large-scale simulation approach capable of teasing out significant relationships and network motifs responsible for producing purely age-driven degenerative phenotypes. Such relationships notably include positive and negative feedback loops, ultrasensitive or switch-like transcriptional responses, and linearity between molecular determinants of aging and pathological cellular phenotypes. More applicably for all biologists (computational or empirical), we discovered that this intuitive simulation construct is capable of making apt predictions in the context of the genetic targets involved. Important genes implicated within this network include *skn-1*, *daf-16*, and *sod-2*, all exceptionally important for controlling the redox state of cells and the integrity of mitochondrial populations.

Many labs continually contribute to the growing body of literature on computational systems biology for mitochondrial aging research. These studies mark the progression toward more simulation-based methods in the field. Although these studies exist at all conceptual scales and levels of abstraction, they all uniquely provide a degree of fruitful quantitative insight, no matter how small [7]. Our group and others have demonstrated the impact of simulation studies for mitochondrial dysfunction on degenerative aging [4–6], drawing upon oxidative signaling as a focal point. As simulations continue to build on one another and expand in their capabilities, we may very well approach a computational tool that the field can consider a realistic representation of mitochondria to unravel the mysteries of biological aging.
